# Serogroup and Clonal Characterization of Czech Invasive *Neisseria meningitidis* Strains Isolated from 1971 to 2015

**DOI:** 10.1371/journal.pone.0167762

**Published:** 2016-12-09

**Authors:** Zuzana Jandova, Martin Musilek, Zuzana Vackova, Jana Kozakova, Pavla Krizova

**Affiliations:** National Reference Laboratory for Meningococcal Infections, National Institute of Public Health, Prague, Czech Republic; Universidad Nacional de la Plata, ARGENTINA

## Abstract

**Background:**

This study presents antigenic and genetic characteristics of *Neisseria meningitidis* strains recovered from invasive meningococcal disease (IMD) in the Czech Republic in 1971–2015.

**Material and Methods:**

A total of 1970 isolates from IMD, referred to the National Reference Laboratory for Meningococcal Infections in 1971–2015, were studied. All isolates were identified and characterized by conventional biochemical and serological tests. Most isolates (82.5%) were characterized by multilocus sequence typing method.

**Results:**

In the study period 1971–2015, the leading serogroup was B (52.4%), most often assigned to clonal complexes cc32, cc41/44, cc18, and cc269. A significant percentage of strains were of serogroup C (41.4%), with high clonal homogeneity due to hyperinvasive complex cc11, which played an important role in IMD in the Czech Republic in the mid-1990s. Serogroup Y isolates, mostly assigned to cc23, and isolates of clonally homogeneous serogroup W have also been recovered more often over the last years.

**Conclusion:**

The incidence of IMD and distribution of serogroups and clonal complexes of *N*. *meningitidis* in the Czech Republic varied over time, as can be seen from the long-term monitoring, including molecular surveillance data. Data from the conventional and molecular IMD surveillance are helpful in refining the antimeningococcal vaccination strategy in the Czech Republic.

## Introduction

Invasive meningococcal disease (IMD) is a global health problem, preventable by vaccination. In view of the great diversity and unique nature of IMD cases, the molecular surveillance which maps the dynamics of the meningococcal populations and detailed characteristics of isolates is of utmost importance for the control of this disease and the development and implementation of effective anti-meningococcal vaccines. In the Czech Republic are available vaccines against all five leading serogroups of *Neisseria meningitidis*: monovalent conjugate vaccine C (MCC), tetravalent conjugate vaccine A, C, W, and Y (MCV4), and four-component vaccine against meningococcus B (4CMenB). This study presents data from molecular characterization of isolates recovered in the Czech Republic over a period of more than four decades. The assignment of IMD isolates to sequence types (ST) and clonal complexes (cc) significantly refines the surveillance of the disease.

On the surface of the bacteria *N*. *meningitidis* is present an antigenic structure, capsular polysaccharide, which is a major factor of virulence in meningococci [1] and a first-line target for humoral immunity. Antigenically different structures have been described in the polysaccharide capsule of meningococci, on the basis of which isolates can be assigned to serogroups. Isolates from IMD are encapsulated and are mostly classified into five serogroups (A, B, C, W and Y) responsible for more than 90% of cases of IMD worldwide [2]. The distribution of causative serogroups varies across the world. While in Africa, the leading serogroups are A, W and X, particularly in the meningitis belt [3], in Europe and Americas, the predominant serogroups were B and C, causing more than 75% of IMD [4,5]. In Europe, IMD cases caused by *N*. *meningitidis* of serogroups Y [6,7] and W [8] are on the rise over the last years.

## Materials and Methods

### Epidemiological data on IMD

IMD is a notifiable disease in the Czech Republic and the incidence data have been available since 1943. The disease was first reported as cerebrospinal and/or meningococcal meningitis. Since 1993, data on IMD cases and deaths have been derived from active surveillance which integrates the epidemiological data reported via the EPIDAT system with the data from the National Reference Laboratory (NRL) for Meningococcal Infections, checked for duplicity in reporting. The IMD case definition used matches the 2012 ECDC case definition, i.e. confirmation of *N*. *meningitidis* from normally sterile site [9].

This study characterizes 1970 isolates from all IMD recovered in the Czech Republic over the period 1971–2015 in terms of serogroups, sequence types, and clonal complexes. *N*. *meningitidis* isolates are referred to the NRL for Meningococcal Infections by clinical microbiology laboratories of the Czech Republic for confirmation and further characterization. As the referral of isolates from IMD to the NRL is in accordance with the Czech law [10], ethical approval and informed consent were not required. The collection of NRL includes over five thousand strains of *N*. *meningitidis* recovered in the Czech Republic since 1971 from various sources: IMD patients, IMD contacts, healthy carriers, patients with respiratory diseases, and rare forms of meningococcal diseases. The database of the collection strains of *N*. *meningitidis* is computerized and clinical, epidemiological, and microbiological data are available for each isolate.

### Identification of *Neisseria meningitidis* and serogrouping

All isolates of *N*. *meningitidis* were identified on the basis of the typical colony morphology and biochemical properties that were first analysed by homemade biochemical tests till the year 1989 (oxidase test and carbohydrate utilization testing for glucose, maltose, sucrose and lactose) and since 1990 by commercial kits API NH test (BioMérieux) or NEISSERIA 4H test (Bio-Rad). Serogrouping was performed by agglutination using commercially available antisera (Bio-Rad, BioMérieux, Oxoid, Murex and Itest).

### Multilocus sequence typing (MLST)

The MLST method was implemented in the NRL for Meningococcal Infections in 2000, since when all isolates from IMD have been prospectively characterized by this method. Recultured IMD isolates since 1971 were tested by MLST retrospectively. MLST was performed as reported by Maiden et al. [11]. Sequencing reactions were analysed on a capillary sequencer ABI PRISM 3130xl (Applied Biosystems), and the data were processed by the Lasergene software (DNASTAR). The results obtained are entered in the national surveillance database and submitted to the following international databases: PubMLST—a multilocus sequence typing database available for *N*. *meningitidis* [12], EMERT—European Meningococcal Epidemiology in Real Time [13], and the ECDC database TESSy—The European Surveillance System [14].

## Results

### Epidemiological situation of IMD and isolates referred to the NRL for Meningococcal Infections

**Fig 1:**
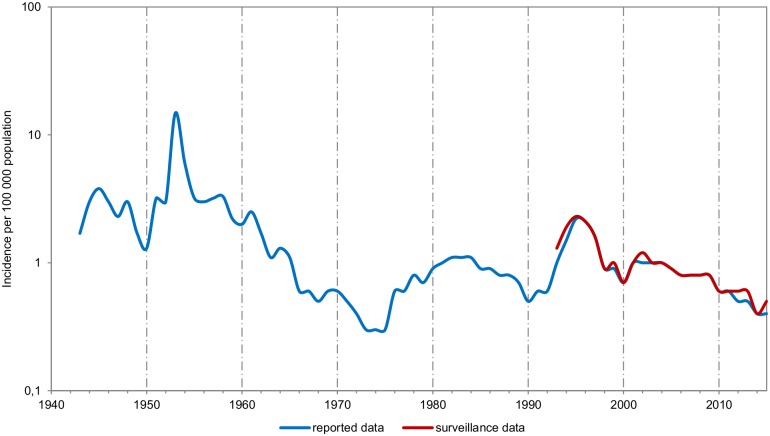
Incidence of invasive meningococcal disease, Czech Republic, 1943–2015. Blue line is incidence (cases per 100 000 population) and the red line is surveillance since 1993.

In the Czech Republic, meningococcal meningitis was on the rise in the 1950s (in 1953, the incidence was 14.8/100 000 population). The increase was followed by a downward trend, with the lowest incidence observed in the 1970s (1973–1975: 0.3/100 000). In the mid-1980s, another increase occurred (peaking in 1982–1984: 1.1/100 000), with a subsequent drop to 0.5/100 000 in 1990. Since 1993, a sharp rise in IMD was observed, peaking to 2.3/100 000 in 1995, followed by a drop without vaccination campaign. Since 2005, the incidence of IMD remains below 1/100 000 and over the last years, it is as low as 0.4 to 0.5/100 000 population (Fig 1).

Since 1993, the average overall case fatality rate for IMD in the Czech Republic was 9.8% and exhibited a stable trend. The case fatality rates varied between years with the leading serogroup causing IMD. Higher case fatality rates were caused by serogroups C, Y, and W.

**Fig 2:**
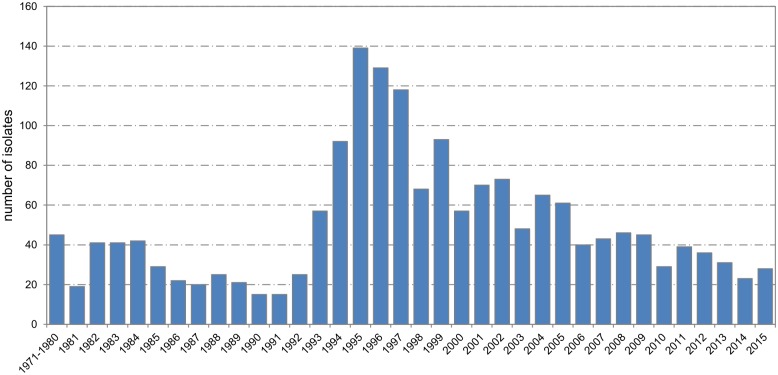
Number of isolates of *N*. *meningitidis* from invasive meningococcal disease, Czech Republic, 1971–2015, n = 1970.

Isolates from IMD have been available since the early 1970s when the laboratory for meningococcal infections, appointed as the National Reference Laboratory in the 1980s, started its activities. Low numbers of isolates were recovered every year until 1992 (Fig 2). This can be explained by two reasons: small numbers of IMD cases and the low percentage (under 40%) of cases from which isolates were referred to the NRL. This situation improved considerably with the start-up of the nation-wide programme of active surveillance in 1993, with the percentage of IMD cases from which isolates were referred to the NRL for confirmation and further characterization reaching over 60% since then. The proportion of the referred IMD isolates is influenced by the fact that about 30% of IMD cases have only been diagnosed by non-culture PCR over the last years [15].

### Serogroups of isolates

**Fig 3:**
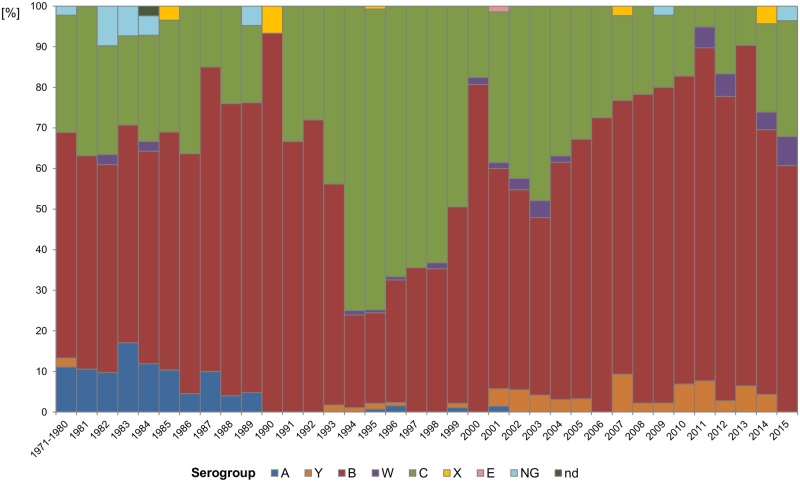
Annual distribution of serogroups of *N*. *meningitidis* from invasive meningococcal disease, Czech republic, 1971–2015, n = 1970 (NG = non groupable, nd = not defined).

Most IMD isolates from the study period were assigned to serogroup B (938 isolates, 52.4%), followed by serogroup C (741 isolates, 41.4%). In individual years, IMD cases were most often caused by serogroup B, with the exception of the 1990s when the leading cause was *N*. *meningitidis* of serogroup C (470 isolates, 59.3%) (Fig 3). Over the last years, serogroup B has become increasingly involved in IMD. Since 2011, serogroup B strains account for 75% of all IMD cases in the Czech Republic. Until 1990, serogroup A was diagnosed as the cause of nearly 9% of IMD cases. Since 2002, no case of IMD was caused by serogroup A in the Czech Republic. Between 1991 and 2010, apart from serogroups B and C (accounting both for 96% of isolates), IMD isolates of serogroups Y (27 isolates, 2.5%) and W (11 isolates, 1%) were also recovered. Over the last five years, equal numbers of cases caused by either *N*. *meningitidis* Y or W have been reported. Other serogroups (X and E) in cumulative terms were recovered from less than 0.5% of all IMD isolates (*N*. *meningitidis* X from five cases and *N*. *meningitidis* E from one case). Thirteen IMD isolates from the study period were classified as *N*. *meningitidis* NG (non-groupable).

### Clonal characterization of isolates

**Fig 4:**
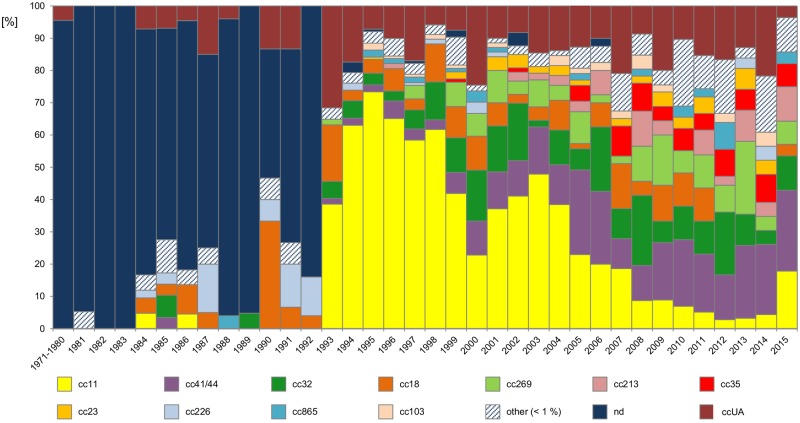
Annual distribution of major clonal complexes of *N*. *meningitidis* from invasive meningococcal disease, Czech Republic, 1971–2015, n = 1970 (cc = clonal complex, ccUA = unassigned clonal complex, nd = not defined).

A total of 1477 isolates (82.5%) were characterized by MLST and assigned to 30 clonal complexes (Fig 4). The most common clonal complexes were cc11 (586 isolates, 32.7%), cc41/44 (142 isolates, 7.9%), cc32 (135 isolates, 7.5%), cc18 (108 isolates, 6%), and cc269 (73 isolates, 4.1%). Other clonal complexes detected were cc213 (30 isolates, 1.7%), cc35 (28 isolates, 1.6%), cc23 (23 isolates, 1.3%), cc226 (20 isolates, 1.1%), cc865 (20 isolates, 1.1%), and cc103 (18 isolates, 1%). Nineteen clonal complexes, whose detection rate was below 1%, are listed in descending order of commonness: cc60, cc8, cc174, cc231, cc162, cc22, cc292, cc116, cc1157, cc167, cc1, cc254, cc37, cc461, cc92, cc106, cc198, cc334, and cc364.

Since 1993, the number of isolates from IMD assigned to clonal complexes has been increasing considerably. Between 1991 and 2000, over half of isolates from IMD (429 isolates, 54.1%) were assigned to cc11. The increase in cc11 was associated with higher IMD incidence caused by serogroup C. Within the same period, certain number of isolates was assigned to cc18 (57 isolates, 7.2%) and cc32 (51 isolates, 6.4%). In 2001–2010, 22 different clonal complexes were detected, with the stagnating predominance of cc11 (144 isolates, 27.7%), followed by cc41/44 (79 isolates, 15.2%), cc32 (63 isolates, 12.1%), and cc269 (39 isolates, 7.5%). In 2011–2015, 19 clonal complexes were detected, with newly predominating cc41/44 (31 isolates, 19.7%), characteristic of serogroup B. Along with increase in cc41/44 over the last five years, cc32 (18 isolates, 11.5%) and cc269 (17 isolates, 10.8%) were on the rise.

As not all strains were available for retrospective testing or were culturable, 313 isolates of *N*. *meningitidis* (17.5%) were not characterised by MLST (nd—not defined). Over 95% of the strains that were not analyzed by MLST had been recovered before 1993 and the last nd isolate was from 2006. Altogether 11.5% (206 isolates) of strains recovered in the course of the study period and investigated by MLST showed ST that did not match any known cc (ccUA—unassigned). The MLST results for isolates not assigned to cc are presented in S1 Table.

The MLST results of nearly all isolates were reported to pubmlst.org database continuously and these results are available there. The MLST results for isolates not referred to this database are presented in S2 Table.

### Serogroup B isolates

**Fig 5:**
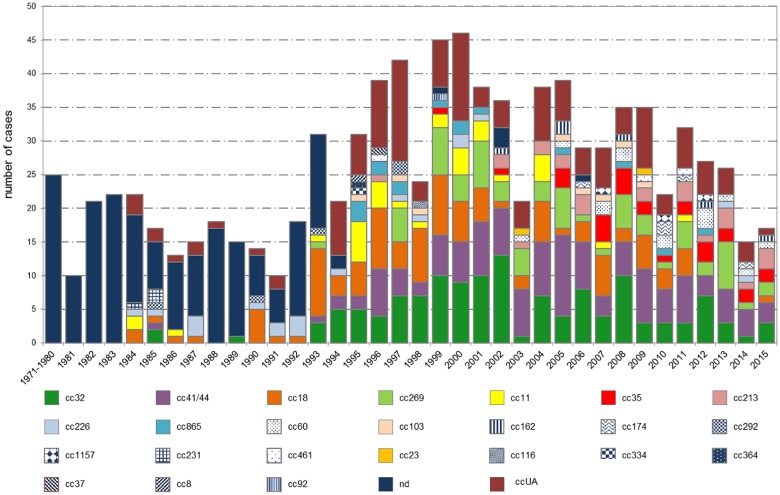
Annual distribution of major clonal complexes of *N*. *meningitidis* B from invasive meningococcal disease, Czech Republic, 1971–2015, n = 938 (cc = clonal complex, ccUA = unassigned clonal complex, nd = not defined).

Serogroup B isolates, that appeared to be the most common and also the most heterogeneous, were assigned to 25 clonal complexes (Fig 5), listed in descending order of commonness: cc32 (133 isolates, 14.2%), cc41/44 (123 isolates, 13.1%), cc18 (105 isolates, 11.2%), and cc269 (67 isolates, 7.1%). Other clonal complexes identified were cc11 (32 isolates, 3.4%), cc35 (27 isolates, 2.9%), cc213 (24 isolates, 2.6%), cc226 (19 isolates, 2%), cc865 (15 isolates, 1.6%), and cc60 (14 isolates, 1.5%). Fourteen clonal complexes, grouped as other in the figure, whose overall detection rate was below 1%, are listed in descending order of commonness: cc103, cc162, cc174, cc292, cc1157, cc231, cc461, cc23, cc116, cc334, cc364, cc37, cc8, and cc92.

Between 1993 and 2000, an increase in cc18 (34 isolates, 19.4%) was observed, with a subsequent significant drop over time (as few as five cases in the last five years). Between 1991 and 2000, apart from the above mentioned cc18, clonal complexes cc32 (50 isolates, 16.3%) and cc41/44 (30 isolates, 9.8%) were revealed more often. In the following period 2001–2010, cc41/44 (70 isolates, 21.7%) prevailed, followed by cc32 (63 isolates, 19.6%) and cc269 along with cc18 (34 isolates, 10.6% each). Over the last five years (2011–2015), cc41/44 accounts for 18.8% (22 isolates) only of all complexes detected. Isolates assigned to cc32 (17 isolates, 14.5%) and cc269 (16 isolates, 13.7%) continue to be rather common. Since 2001 cc18 has been declining, while cc35 and cc213 have been revealed more often.

### Serogroup C isolates

**Fig 6:**
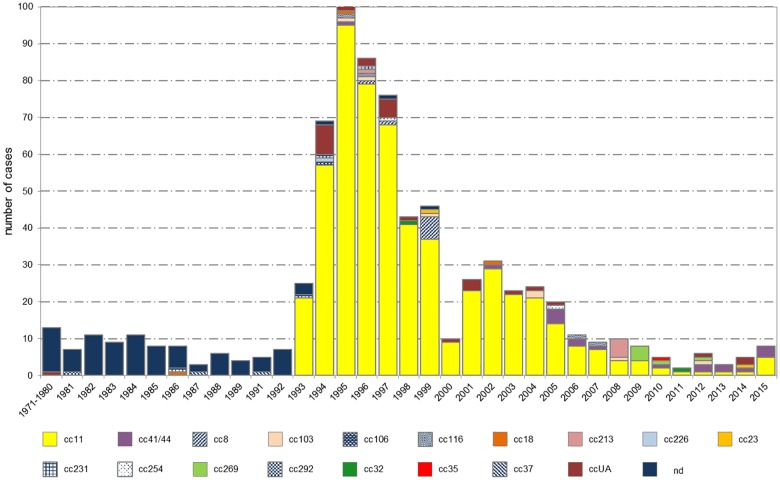
Annual distribution of major clonal complexes of *N*. *meningitidis* C from invasive meningococcal disease, Czech Republic, 1971–2015, n = 741 (cc = clonal complex, ccUA = unassigned clonal complex, nd = not defined).

Despite spanning 18 clonal complexes, serogroup C appeared to be clonally more homogeneous compared to serogroup B, considering the fact, that nearly three quarters (550 isolates, 74.2%) of all cases of IMD caused by *N*. *meningitidis* C were assigned to cc11. This clonal complex prevailed from 1993 to 2015, with the exception of 2012 and 2013, when the leading clonal complex of serogroup C was cc41/44 (Fig 6). The leading cc11 was followed by cc41/44 (18 isolates, 2.4%) and cc8 (8 isolates, 1.1%). Over the last five years, comparable rates of cases were caused by cc41/44 (8 isolates, 33.3%) and cc11 (9 isolates, 37.5%). Fourteen clonal complexes, grouped as other in the figure, whose overall detection rate was below 1%, are listed in descending order of commonness: cc103, cc213, cc269, cc116, cc231, cc18, cc23, cc254, cc32, cc37, cc106, cc226, cc292, and cc35.

### Isolates of serogroups A, Y, and W

Cases of IMD caused by serogroup A (36 isolates, 2.01%) were reported early in the study period, for the last time in 2001. The isolates were assigned to clonal complexes cc1, cc103, and cc865 (Table 1). Strains of cc1 showed typical characteristics of serogroup A. As serogroup A strains occurred previously and were not culturable, 29 isolates (81%) could not be assigned retrospectively to clonal complex. The leading clonal complex of serogroup Y (35 isolates, 1.96%) was cc23 (18 isolates, 51.4%). Other rather common clonal complexes were cc167, cc103, and previously found cc92. Over one quarter of *N*. *meningitidis* isolates of serogroup Y showed ST unassigned to cc (ccUA, 26.5%). The fifth leading serogroup causing IMD in the Czech Republic was W (20 isolates, 1.12%). Serogroup W was rather homogeneous in terms of clonal complexes, with cc22 (6 isolates, 30%) as the leading clonal complex.

**Table 1:** Clonal characterization of meningococcal isolates of *N*. *meningitidis* A, Y, and W from invasive meningococcal disease, Czech Republic, 1971–2015, n = 60 = investigated by MLST.

	Serogroup A (n = 7)			Serogroup Y (n = 34)				Serogroup W (n = 19)			
Clonal complex (cc)	cc1	cc103	cc865	cc23	cc167	cc92	cc103	cc22	cc865	cc11	cc174
**Number of isolates**	3	2	1	18	4	2	1	6	4	4	3
**% in the serogroup**	42.9	28.6	14.3	52.9	11.8	5.9	2.9	31.6	21.1	21.1	15.8
**Specificity for the serogroup**	yes	no	no	yes	yes	yes	no	yes	no	no	no
**ccUA (%)**	1 (14.3)			9 (26.5)				2 (10.5)			

Serogroups A, Y, and W, are after serogroups B and C most common in Czech Republic, (ccUA = unassigned clonal complex).

## Discussion

Most cases of IMD are caused by hyperinvasive clones, stable over time, assigned to specific clonal complexes defined by MLST [11]. It was published that some clonal complexes are common, and their incidence does not vary with time or place [16]. Some studies have reported a significant incidence of specific clonal complexes in specific serogroups of *N*. *meningitidis*: cc41/44 and cc32 are typical of serogroup B, cc11 is typical of serogroup C, and cc23 is typical of serogroup Y [17–20]. Our results are consistent with these findings.

In the last years, IMD in the Czech Republic is most commonly caused by serogroup B which accounts for up to 70% of all cases. Serogroup B strains show high clonal variability in both, IMD and healthy carriers isolates, as evidenced by our other study [21]. The source data in cited publication are subset data of this study. The first study was focused to the comparison of IMD and healthy carriers isolates, while the recent study presented in this paper is focused to IMD isolates only, but in more detailed and complex analysis. As reported from other countries, the serogroup B meningococcal population is more diverse in comparison with serogroup C [5].

The incidence of the most common clonal complexes in the Czech Republic is in general agreement with that in Europe [22]. In the study referred to above, cc41/44 from serogroup B and cc11 typical of *N*. *meningitidis* serogroup C were most often recovered in 18 European countries over a three-year period. The following most common clonal complexes were cc32, cc8, and cc269. Unlike in the study referred to above, cc8 is rarely found in the Czech Republic and was more common only late in the last century. Similar data on the detection of the above-mentioned clonal complexes among *N*. *meningitidis* B isolates have been reported in neighbouring Poland, with cc32, cc18, and cc41/44 being the most common [23]. In the Czech Republic, cc18 has been recovered since 1984 and was predominant in the 1990s. The clonal distribution of the Czech IMD isolates of serogroup B correlates with the data from England and Wales where the four most common clonal complexes of *N*. *meningitidis* B in 2006–2009 were cc269, cc41/44, cc213, and cc32 [24]. The clonal complexes cc32 and cc41/44 are comparably common in the Czech Republic. The leading clonal complex from the UK, cc269, is more commonly recovered in the Czech Republic over the last years as well (10.8% in 2011–2015). IMD isolates of cc213 are also on the rise over the last years, with those from 2011–2015 accounting for 36.7% of all cc213 isolates detected in the Czech Republic. In both the UK and the Czech Republic, the leading serogroup of *N*. *meningitidis* isolates from IMD is B, followed by serogroup Y in the UK, while in the Czech Republic, the second leading serogroup is C, followed by serogroup Y, which is also on the rise.

One of the reasons for the high proportion of IMD being caused by serogroup B (reaching at times over 75%) in many countries is the impact of mass vaccination against *N*. *meningitidis* C [25–27]. However, in some countries including the Czech Republic, serogroup B is prevailing, although the MenC vaccine is not used for large-scale immunisation. Serogroup C meningococci were responsible for increase in IMD cases in the Czech Republic in the mid-1980s and 1990s. The serogroup involved in cases of meningococcal meningitis in the Czech Republic in the 1950s remains unidentified, as isolates from patients from that period are not available. The increase in IMD cases in the mid-1980s was caused by the above-mentioned serogroup C, and additional molecular characterization of isolates showed that the cases were due to local *N*. *meningitidis* C strains, not belonging to cc11. The sharp rise in IMD cases in the mid-1990s was caused by *N*. *meningitidis* C cc11, which was not detected in the Czech Republic since the 1970s and thus was completely new to the Czech population. In the mid-1990, conjugate vaccine against *N*. *meningitidis* was not available in the Czech Republic. When marketed in 2001, IMD cases caused by serogroup C were declining and therefore mass vaccination against serogroup C meningococci was not implemented.

Increase in IMD cases caused by *N*. *meningitidis* C was reported in Poland since 2002 [23]. The most common clonal complexes involved in IMD were cc8 and cc103, not found in Poland before. These clonal complexes were also identified among IMD cases caused by serogroup C in the Czech Republic: cc8 is rather rare and was detected mainly at the end of the last millennium and cc103 is the fourth leading clonal complex of *N*. *meningitidis* C in the country. In Poland, *N*. *meningitidis* C isolates of cc103 were the most common in 2005 and then since 2008, representing up to 42% of all isolates of this serogroup [23]. The third leading clonal complex of *N*. *meningitidis* C in Poland is cc11. Among meningococci of serogroup C, cc11 appears to be a hyperinvasive clonal complex responsible for increase in IMD [28]. It is a globally spread clonal complex associated not only with strains of serogroup C but also of other serogroups. Most IMD cases caused by serogroup C in North America, Europe and Africa are due to cc11 known to be the cause of both endemic and pandemic cases in different parts of the world [29].

Czech invasive isolates of *N*. *meningitidis* Y cc23 have been reported since 1995 and account for 51% of all isolates of this serogroup. Similar increasing trends were observed in other European countries such as Sweden [30] and the UK [6] or in the USA [31]. IMD cases caused by *N*. *meningitidis* Y cc23 were on the rise in the USA in the 1990s [32]. In Europe, an increasing incidence of IMD caused by meningococci of serogroup Y has been reported e.g. in Italy where the causative strains of this serogroup, predominantly of cc23, have been of the rise since 2008 [33].

Since 2002, IMD cases caused by serogroup A have not been reported in the Czech Republic. A similar situation has been observed in most European countries. However, serogroup A was and continues to be responsible for the majority of meningococcal outbreaks in the African meningitis belt [34]. Although the introduction of the monovalent conjugate vaccine against *N*. *meningitidis* A in some African regions resulted in the reduction of serogroup A cases and transmission [35], serogroup A continues to be the cause of most recent outbreaks. The leading clonal complex in these cases is cc5 [36], which differs from the clonal complexes previously detected in the Czech Republic for this serogroup.

Isolates of serogroup W have been regularly, but not frequently, identified in the Czech Republic. Thirty percent of serogroup W isolates were assigned to cc22. In general, cc22 typically show serogroup W characteristics. An over 50% incidence of cc22 in the above-mentioned serogroup has been reported by a UK study [37]. Strains of serogroup W, cc11 are characterised by a high case fatality rate [38]. Serogroup W strains of cc11 are also the cause of IMD cases in the Middle East, associated with the annual Muslim pilgrimages to Saudi Arabia [39]. In the Czech Republic, cc11 and cc174 were previously found among serogroup W strains (particularly in the 1990s). The area where IMD cases caused by serogroup W are on the rise is still the meningitis belt in Africa. Serogroup W strains appear to be the leading cause of IMD in Africa over the last 15 years [40]. Thanks also to a good preventive control of *N*. *meningitidis* of serogroup A, serogroup W strains have recently become the most prevalent cause of the disease [35].

Results of this study show that the incidence of IMD and genetic and antigenic characteristics of isolates from IMD in the Czech Republic vary over time. IMD is rather rare, but its severity and a high case fatality rate are a challenge for the implementation of preventive measures, the most effective of which is vaccination. Data from conventional and molecular surveillance of IMD provide a background for refining the anti-meningococcal vaccination strategy in the Czech Republic [41]. In cooperation with the NRL and the Czech Vaccinology Association, there were produced the Guidelines on immunisation against invasive meningococcal disease, approved by the National Immunisation Committee (NICO) and available at the NRL website [42]. This recommendation emphasizes the need for a long-term and broad protection of individuals and in particular of those at high risk of IMD.

## Conclusions

The analysis of the distribution of serogroups and clonal complexes of *N*. *meningitidis* causing IMD in the Czech Republic in 1971–2015 shows *N*. *meningitidis* of serogroup B, the most prevalent serogroup over almost the whole study period, to be typical for this country, with the exception of the mid-1990s characterized by the prevalence of *N*. *meningitidis* strains of serogroup C, the emergence of which always caused increase in IMD cases and in the case fatality rate. Over the whole study period, serogroup B strains show a high heterogeneity and variability of clonal complexes (with the prevalence of cc32, cc41/44, cc18, and cc269), while serogroup C is clonally homogeneous, with cc11, first reported in the Czech Republic in 1993, being the most common. *N*. *meningitidis* strains of serogroups A, Y, and W rarely cause IMD in the Czech Republic and have different dynamics over time: serogroup A emerged early in the study period while serogroups Y and W were observed much later. Serogroup A strains were mostly assigned to typical cc1, the leading clonal complex of serogroup Y was cc23, and cc22 was often found in serogroup W. Data from IMD surveillance, including molecular data, provide a background for updating the vaccination strategy in the Czech Republic.

## Supporting Information

S1 Table All isolates from the study with no clonal complex (ccUA). (PDF)

S2 Table The isolates from the study not referred to pubmlst.org database. (PDF)
