# Supplementary material for: Serogroup and Clonal Characterization of Czech Invasive Neisseria meningitidis Strains Isolated from 1971 to 2015
Source: PLoS One. 2016 Dec 9;11(12):e0167762. doi: 10.1371/journal.pone.0167762 (PMC5147975; doi:10.1371/journal.pone.0167762)
Supplement: S1 Table — (PDF) [file pone.0167762.s001.pdf]

S1 Table. All isolates from the study with no clonal complex (ccUA)

ID of isolate	Year of isolation	Serogroup	Sequence type (ST)	Allelic profile							Clonal complex (cc)
				abcZ	adk	aroE	fumC	gdh	pdhC	pgm	
0304/77	1977	Y	4743	180	133	2	343	217	204	8	ccUA
0189/80	1980	C	101	6	6	9	1	26	6	32	ccUA
0049/84	1984	B	1012	12	2	4	8	13	17	78	ccUA
0080/84	1984	B	120	12	17	6	39	9	22	8	ccUA
0081/84	1984	B	501	9	5	6	5	6	8	9	ccUA
0879/85	1985	B	4745	12	7	4	36	5	21	16	ccUA
0933/85	1985	B	130	23	5	9	3	13	32	8	ccUA
0070/86	1986	B	539	9	5	9	17	79	6	83	ccUA
0021/87	1987	A	557	57	5	2	9	6	68	12	ccUA
0076/87	1987	B	130	23	5	9	3	13	32	8	ccUA
0081/87	1987	B	132	24	8	10	19	25	35	33	ccUA
0024/88	1988	B	559	6	6	6	1	26	6	32	ccUA
0018/90	1990	B	5003	9	5	6	143	5	119	18	ccUA
0037/90	1990	X	1220	2	18	15	55	24	11	10	ccUA
0055/91	1991	B	4754	12	8	15	26	10	6	16	ccUA
0059/91	1991	B	4754	12	8	15	26	10	6	16	ccUA
0001/93	1993	B	668	2	2	9	36	17	106	51	ccUA
0003/93	1993	C	678	6	6	9	1	26	6	110	ccUA
0005/93	1993	B	1019	20	2	9	131	5	11	9	ccUA
0007/93	1993	B	718	63	17	25	1	11	21	2	ccUA
0012/93	1993	B	86	2	18	6	40	26	6	1	ccUA

0050/93	1993	B	732	1	2	6	13	58	108	20	ccUA
0181/93	1993	C	678	6	6	9	1	26	6	110	ccUA
0184/93	1993	B	669	2	2	9	36	9	6	51	ccUA
0192/93	1993	B	996	2	3	15	40	3	105	9	ccUA
0282/93	1993	B	36	12	7	4	8	5	21	16	ccUA
0283/93	1993	Y	36	12	7	4	8	5	21	16	ccUA
0347/93	1993	B	698	12	18	112	110	58	109	27	ccUA
0390/93	1993	C	101	6	6	9	1	26	6	32	ccUA
0443/93	1993	B	669	2	2	9	36	9	6	51	ccUA
0483/93	1993	B	698	12	18	112	110	58	109	27	ccUA
0497/93	1993	B	742	12	31	6	39	9	22	8	ccUA
0503/93	1993	B	117	12	8	15	26	10	1	16	ccUA
0517/93	1993	B	704	16	2	9	36	40	6	21	ccUA
0077/94	1994	C	678	6	6	9	1	26	6	110	ccUA
0078/94	1994	B	130	23	5	9	3	13	32	8	ccUA
0081/94	1994	C	101	6	6	9	1	26	6	32	ccUA
0083/94	1994	B	2018	12	6	15	17	58	21	27	ccUA
0092/94	1994	B	2019	7	6	32	5	10	11	2	ccUA
0109/94	1994	C	130	23	5	9	3	13	32	8	ccUA
0184/94	1994	B	130	23	5	9	3	13	32	8	ccUA
0201/94	1994	C	6084	402	3	19	17	62	21	2	ccUA
0204/94	1994	C	678	6	6	9	1	26	6	110	ccUA
0253/94	1994	B	117	12	8	15	26	10	1	16	ccUA
0262/94	1994	C	101	6	6	9	1	26	6	32	ccUA
0309/94	1994	B	2021	12	8	39	5	10	6	9	ccUA
0380/94	1994	B	36	12	7	4	8	5	21	16	ccUA
0386/94	1994	B	120	12	17	6	39	9	22	8	ccUA
0533/94	1994	C	2022	6	6	9	16	26	6	58	ccUA
0550/94	1994	C	125	20	5	7	35	6	30	12	ccUA
0020/95	1995	B	6400	12	8	18	39	9	22	8	ccUA
0037/95	1995	C	678	6	6	9	1	26	6	110	ccUA
0103/95	1995	C	101	6	6	9	1	26	6	32	ccUA
0109/95	1995	C	101	6	6	9	1	26	6	32	ccUA
0115/95	1995	B	5920	20	7	15	25	50	11	21	ccUA

0120/95	1995	X	117	12	8	15	26	10	1	16	ccUA
0267/95	1995	B	6401	12	3	4	8	392	21	78	ccUA
0348/95	1995	B	669	2	2	9	36	9	6	51	ccUA
0477/95	1995	B	1290	58	6	52	2	26	17	9	ccUA
0479/95	1995	B	6097	12	17	6	39	9	418	424	ccUA
0006/96	1996	B	2766	12	17	4	39	9	22	8	ccUA
0008/96	1996	Y	130	23	5	9	3	13	32	8	ccUA
0026/96	1996	B	1883	6	5	4	1	26	6	32	ccUA
0029/96	1996	B	101	6	6	9	1	26	6	32	ccUA
0096/96	1996	B	2024	2	18	6	1	26	6	1	ccUA
0175/96	1996	C	678	6	6	9	1	26	6	110	ccUA
0195/96	1996	B	144	7	17	32	5	10	11	2	ccUA
0239/96	1996	B	5925	12	5	2	28	8	86	139	ccUA
0247/96	1996	B	4761	24	8	10	19	25	35	333	ccUA
0289/96	1996	B	4955	12	3	166	11	242	40	238	ccUA
0293/96	1996	B	5926	12	245	4	72	3	21	139	ccUA
0441/96	1996	B	130	23	5	9	3	13	32	8	ccUA
0468/96	1996	C	678	6	6	9	1	26	6	110	ccUA
0007/97	1997	B	147	12	17	33	39	9	22	2	ccUA
0041/97	1997	B	6086	165	18	293	3	9	139	21	ccUA
0053/97	1997	B	1377	8	17	25	37	5	21	16	ccUA
0071/97	1997	B	5927	2	18	9	40	26	6	1	ccUA
0072/97	1997	B	5928	20	6	6	2	6	5	8	ccUA
0080/97	1997	B	1274	9	7	9	133	9	33	8	ccUA
0086/97	1997	C	101	6	6	9	1	26	6	32	ccUA
0136/97	1997	B	6099	12	18	15	17	58	21	425	ccUA
0178/97	1997	B	1377	8	17	25	37	5	21	16	ccUA
0196/97	1997	C	678	6	6	9	1	26	6	110	ccUA
0203/97	1997	B	1274	9	7	9	133	9	33	8	ccUA
0212/97	1997	C	6087	403	6	9	1	26	6	32	ccUA
0223/97	1997	B	4955	12	3	166	11	242	40	238	ccUA
0224/97	1997	B	120	12	17	6	39	9	22	8	ccUA
0225/97	1997	B	120	12	17	6	39	9	22	8	ccUA
0282/97	1997	B	1836	12	8	188	14	10	1	16	ccUA

0303/97	1997	B	1836	12	8	188	17	10	1	16	ccUA
0308/97	1997	C	6087	403	6	9	1	26	6	32	ccUA
0322/97	1997	B	4955	12	3	166	11	242	40	238	ccUA
0330/97	1997	C	5238	6	5	393	17	21	24	2	ccUA
0001/98	1998	B	669	2	2	9	36	9	6	51	ccUA
0066/98	1998	B	2177	7	106	10	17	10	6	9	ccUA
0138/98	1998	C	678	6	6	9	1	26	6	110	ccUA
0146/98	1998	B	669	2	2	9	36	9	6	51	ccUA
0033/99	1999	B	2177	7	106	10	17	10	6	9	ccUA
0034/99	1999	B	117	12	8	15	26	10	1	16	ccUA
0107/99	1999	B	939	20	5	75	58	1	21	20	ccUA
0147/99	1999	B	239	7	4	10	26	10	18	16	ccUA
0198/99	1999	B	*939	20	5	75	*58	1	21	20	ccUA
0208/99	1999	B	875	8	17	25	35	5	21	16	ccUA
0296/99	1999	B	5930	21	5	6	5	6	8	2	ccUA
0003/00	2000	B	3475	4	3	2	17	5	11	20	ccUA
0013/00	2000	B	939	20	5	75	58	1	21	20	ccUA
0035/00	2000	B	130	23	5	9	3	13	32	8	ccUA
0039/00	2000	B	215	9	6	45	35	8	11	2	ccUA
0041/00	2000	B	3019	12	17	269	34	23	34	28	ccUA
0042/00	2000	B	5134	24	8	10	19	25	35	10	ccUA
0045/00	2000	B	5134	24	8	10	19	25	35	10	ccUA
0059/00	2000	C	101	6	6	9	1	26	6	32	ccUA
0068/00	2000	B	5134	24	8	10	26	25	35	10	ccUA
0073/00	2000	B	939	20	5	75	58	1	21	20	ccUA
0074/00	2000	B	5134	24	8	10	19	25	*	10	ccUA
0106/00	2000	B	2177	7	106	10	17	10	6	9	ccUA
0135/00	2000	B	939	20	5	75	58	1	21	20	ccUA
0141/00	2000	B	2991	8	4	6	40	257	246	16	ccUA
0022/01	2001	B	3697	63	17	25	119	11	21	2	ccUA
0024/01	2001	C	3698	7	106	10	17	10	13	9	ccUA
0033/01	2001	B	2177	7	106	10	17	10	6	9	ccUA
0107/01	2001	W	1184	46	20	4	7	58	20	8	ccUA
0143/01	2001	B	875	8	17	25	35	5	21	16	ccUA

0176/01	2001	C	2015	8	121	15	26	10	1	16	ccUA
0231/01	2001	C	2015	8	121	15	26	10	1	16	ccUA
0003/02	2002	B	232	2	5	9	9	9	18	8	ccUA
0087/02	2002	Y	2993	23	5	34	9	13	32	8	ccUA
0102/02	2002	B	2991	8	4	6	40	257	246	16	ccUA
0151/02	2002	B	2494	8	5	9	9	9	18	8	ccUA
0170/02	2002	W	3889	23	5	9	3	8	32	8	ccUA
0179/02	2002	B	3906	20	5	75	3	1	21	20	ccUA
0038/03	2003	C	939	20	5	75	58	1	21	20	ccUA
0066/03	2003	Y	130	23	5	9	3	13	32	8	ccUA
0121/03	2003	B	5931	2	2	9	1	9	6	51	ccUA
0148/03	2003	B	3352	12	7	4	266	5	21	16	ccUA
0157/03	2003	Y	3015	219	5	275	17	11	8	21	ccUA
0167/03	2003	B	5932	9	16	4	17	9	11	44	ccUA
0184/03	2003	B	1274	9	7	9	133	9	33	8	ccUA
0001/04	2004	B	5131	12	2	33	39	9	22	2	ccUA
0039/04	2004	B	5032	7	8	4	26	10	11	8	ccUA
0098/04	2004	B	5132	12	92	6	39	9	22	8	ccUA
0124/04	2004	C	4781	12	4	15	110	58	6	27	ccUA
0163/04	2004	B	4782	2	5	9	36	9	177	18	ccUA
0174/04	2004	B	4783	2	5	9	110	9	18	8	ccUA
0178/04	2004	B	5760	8	18	6	40	26	387	1	ccUA
0259/04	2004	B	2177	7	106	10	17	10	6	9	ccUA
0295/04	2004	B	4785	7	2	10	26	10	333	16	ccUA
0065/05	2005	Y	3015	219	5	275	17	11	8	21	ccUA
0093/05	2005	B	36	12	7	4	8	5	21	16	ccUA
0109/05	2005	B	919	12	5	139	16	8	13	62	ccUA
0118/05	2005	B	669	2	2	9	36	9	6	51	ccUA
0159/05	2005	B	4954	6	5	105	12	6	13	17	ccUA
0163/05	2005	C	5238	6	5	393	17	21	24	2	ccUA
0171/05	2005	B	5126	10	6	63	199	9	6	12	ccUA
0284/05	2005	B	5129	2	5	9	36	9	6	40	ccUA
0056/06	2006	B	669	2	2	9	36	9	6	51	ccUA
0092/06	2006	B	669	2	2	9	36	9	6	51	ccUA

0101/06	2006	B	5581	2	5	9	343	9	373	8	ccUA
0168/06	2006	B	5897	2	5	9	36	9	6	51	ccUA
0004/07	2007	B	6089	4	3	2	17	8	11	8	ccUA
0049/07	2007	B	5937	12	5	9	54	8	21	20	ccUA
0058/07	2007	B	85	2	5	9	36	9	6	18	ccUA
0105/07	2007	Y	130	23	5	9	3	13	32	8	ccUA
0113/07	2007	B	669	2	2	9	36	9	6	51	ccUA
0114/07	2007	X	90	2	18	27	37	24	11	10	ccUA
0121/07	2007	B	6330	2	5	9	9	9	21	8	ccUA
0152/07	2007	B	6396	9	6	72	58	5	11	347	ccUA
0156/07	2007	Y	3015	219	5	275	17	11	8	21	ccUA
0009/08	2008	B	2917	12	17	6	3	9	22	8	ccUA
0046/08	2008	B	875	8	17	25	35	5	21	16	ccUA
0070/08	2008	B	6587	12	6	4	35	13	437	446	ccUA
0116/08	2008	B	6760	20	5	10	3	10	442	16	ccUA
0023/09	2009	B	2177	7	106	10	17	10	6	9	ccUA
0045/09	2009	B	232	2	5	9	9	9	18	8	ccUA
0046/09	2009	B	232	2	5	9	9	9	18	8	ccUA
0108/09	2009	B	7641	4	8	523	26	10	1	16	ccUA
0110/09	2009	B	7642	12	17	6	35	9	22	8	ccUA
0115/09	2009	B	232	2	5	9	9	9	18	8	ccUA
0119/09	2009	B	132	24	8	10	19	25	35	33	ccUA
0148/09	2009	B	704	16	2	9	36	40	6	21	ccUA
0003/10	2009	B	232	2	5	9	9	9	18	8	ccUA
0030/10	2010	B	132	23	8	10	19	25	35	33	ccUA
0035/10	2010	B	232	2	5	9	9	9	18	8	ccUA
0058/10	2010	B	8506	4	5	6	17	8	31	8	ccUA
0011/11	2011	B	85	2	5	9	36	9	6	18	ccUA
0021/11	2011	B	17	8	3	13	1	11	12	4	ccUA
0022/11	2011	B	232	2	5	9	9	9	18	8	ccUA
0068/11	2011	B	8976	12	5	15	54	8	588	20	ccUA
0078/11	2011	B	9086	4	6	2	5	607	11	8	ccUA
0094/11	2011	B	130	23	5	9	3	13	32	8	ccUA
0007/12	2012	C	9301	7	8	4	26	10	11	6	ccUA

0012/12	2012	B	9303	576	17	9	35	5	21	16	ccUA
0037/12	2012	B	85	2	5	9	36	9	6	18	ccUA
0040/12	2012	B	9471	8	8	9	26	10	1	9	ccUA
0074/12	2012	B	6330	2	5	9	9	9	21	8	ccUA
0083/12	2012	B	10006	2	2	9	36	9	648	51	ccUA
0001/13	2013	B	939	20	5	75	58	1	21	20	ccUA
0004/13	2013	B	232	2	5	9	9	9	18	8	ccUA
0036/13	2013	B	669	2	2	9	36	9	6	51	ccUA
0046/13	2013	B	10298	12	17	6	17	10	22	685	ccUA
0013/14	2014	B	232	2	5	9	9	9	18	8	ccUA
0032/14	2014	B	7148	6	7	7	451	21	18	17	ccUA
0036/14	2014	C	5321	6	41	2	359	50	129	15	ccUA
0038/14	2014	C	10909	12	5	4	9	5	6	9	ccUA
0069/14	2014	B	11049	9	60	6	9	9	11	16	ccUA
0067/15	2015	B	11532	12	17	6	9	9	22	8	ccUA
