# Supplementary material for: Serogroup and Clonal Characterization of Czech Invasive Neisseria meningitidis Strains Isolated from 1971 to 2015
Source: PLoS One. 2016 Dec 9;11(12):e0167762. doi: 10.1371/journal.pone.0167762 (PMC5147975; doi:10.1371/journal.pone.0167762)
Supplement: S2 Table — (PDF) [file pone.0167762.s002.pdf]

S2 Table. The isolates from the study not referred to pubmlst.org database

ID of isolate	Year of isolation	Serogroup	Sequence type (ST)	Clonal complex (cc)
0056/84	1984	B	236	cc18
0057/84	1984	B	226	cc226
0080/84	1984	B	120	ccUA
0081/84	1984	B	501	ccUA
0613/84	1984	B	231	cc231
0015/85	1985	B	292	cc292
0878/85	1985	B	33	cc32
0892/85	1985	B	44	cc41/44
0895/85	1985	B	226	cc226
0933/85	1985	B	130	ccUA
0934/85	1985	B	32	cc32
0052/86	1986	B	11	cc11
0059/86	1986	C	231	cc231
0044/87	1987	B	226	cc226
0045/87	1987	B	226	cc226
0066/87	1987	C	697	cc37
0076/87	1987	B	130	ccUA
0081/87	1987	B	132	ccUA
0002/90	1990	B	18	cc18
0018/90	1990	B	5003	ccUA
0036/90	1990	B	145	cc18
0042/90	1990	B	145	cc18
0059/91	1991	B	4754	ccUA
0014/92	1992	B	4756	cc18
0023/92	1992	B	226	cc226
0035/92	1992	B	226	cc226
0348/93	1993	B	659	cc18
0540/93	1993	C	11	cc11
0042/94	1994	C	11	cc11
0076/94	1994	C	11	cc11
0078/94	1994	B	130	ccUA
0081/94	1994	C	101	ccUA
0093/94	1994	C	11	cc11
0109/94	1994	C	130	ccUA
0113/94	1994	C	11	cc11
0131/94	1994	C	11	cc11
0144/94	1994	C	11	cc11
0181/94	1994	B	226	cc226
0199/94	1994	C	11	cc11
0245/94	1994	C	11	cc11
0250/94	1994	C	11	cc11
0256/94	1994	C	11	cc11
0258/94	1994	C	11	cc11
0262/94	1994	C	101	ccUA
0263/94	1994	C	11	cc11

0265/94	1994	C	11	cc11
0272/94	1994	C	11	cc11
0279/94	1994	C	11	cc11
0284/94	1994	C	11	cc11
0289/94	1994	C	11	cc11
0301/94	1994	C	11	cc11
0313/94	1994	C	11	cc11
0336/94	1994	C	11	cc11
0340/94	1994	B	1015	cc32
0342/94	1994	C	11	cc11
0345/94	1994	C	11	cc11
0355/94	1994	C	11	cc11
0360/94	1994	C	11	cc11
0362/94	1994	C	11	cc11
0382/94	1994	C	11	cc11
0385/94	1994	C	11	cc11
0386/94	1994	B	120	ccUA
0395/94	1994	C	11	cc11
0397/94	1994	C	11	cc11
0504/94	1994	C	11	cc11
0517/94	1994	C	11	cc11
0529/94	1994	C	11	cc11
0531/94	1994	B	32	cc32
0532/94	1994	C	11	cc11
0534/94	1994	C	11	cc11
0535/94	1994	B	1915	cc41/44
0537/94	1994	C	11	cc11
0538/94	1994	C	11	cc11
0547/94	1994	B	720	cc18
0549/94	1994	B	136	cc41/44
0551/94	1994	C	11	cc11
0553/94	1994	B	32	cc32
0560/94	1994	C	11	cc11
0001/95	1994	C	11	cc11
0003/95	1994	C	11	cc11
0004/95	1994	B	1015	cc32
0007/95	1994	C	11	cc11
0008/95	1994	B	1015	cc32
0009/95	1995	C	11	cc11
0010/95	1995	C	11	cc11
0017/95	1995	C	11	cc11
0018/95	1995	C	18	cc18
0022/95	1995	C	11	cc11
0023/95	1995	C	11	cc11
0024/95	1995	C	11	cc11
0030/95	1995	C	11	cc11
0032/95	1995	C	11	cc11
0033/95	1995	C	11	cc11
0034/95	1995	C	11	cc11
0035/95	1995	C	11	cc11
0036/95	1995	B	103	cc103
0037/95	1995	C	678	ccUA

0040/95	1995	Y	569	cc23
0041/95	1995	C	11	cc11
0050/95	1995	C	11	cc11
0052/95	1995	C	11	cc11
0066/95	1995	C	11	cc11
0102/95	1995	B	11	cc11
0103/95	1995	C	101	ccUA
0104/95	1995	C	11	cc11
0105/95	1995	C	11	cc11
0106/95	1995	C	11	cc11
0109/95	1995	C	101	ccUA
0110/95	1995	C	11	cc11
0111/95	1995	C	11	cc11
0112/95	1995	B	112	cc41/44
0113/95	1995	C	11	cc11
0122/95	1995	C	11	cc11
0128/95	1995	C	11	cc11
0132/95	1995	C	11	cc11
0133/95	1995	C	11	cc11
0141/95	1995	C	11	cc11
0144/95	1995	C	11	cc11
0146/95	1995	C	11	cc11
0148/95	1995	C	11	cc11
0206/95	1995	C	11	cc11
0207/95	1995	C	11	cc11
0212/95	1995	C	11	cc11
0218/95	1995	C	11	cc11
0222/95	1995	B	18	cc18
0255/95	1995	C	11	cc11
0256/95	1995	B	225	cc865
0258/95	1995	C	11	cc11
0260/95	1995	C	11	cc11
0264/95	1995	C	11	cc11
0265/95	1995	C	11	cc11
0266/95	1995	C	11	cc11
0269/95	1995	C	11	cc11
0275/95	1995	C	11	cc11
0281/95	1995	C	11	cc11
0302/95	1995	C	11	cc11
0306/95	1995	C	11	cc11
0308/95	1995	B	112	cc41/44
0309/95	1995	C	11	cc11
0310/95	1995	C	11	cc11
0315/95	1995	C	11	cc11
0317/95	1995	C	11	cc11
0318/95	1995	W	11	cc11
0322/95	1995	C	11	cc11
0332/95	1995	C	11	cc11
0340/95	1995	A	103	cc103
0342/95	1995	C	11	cc11
0344/95	1995	C	11	cc11
0345/95	1995	C	11	cc11

0347/95	1995	C	11	cc11
0348/95	1995	B	669	ccUA
0350/95	1995	C	11	cc11
0352/95	1995	C	11	cc11
0359/95	1995	C	11	cc11
0360/95	1995	C	11	cc11
0361/95	1995	C	11	cc11
0362/95	1995	C	11	cc11
0365/95	1995	C	11	cc11
0371/95	1995	C	11	cc11
0372/95	1995	B	118	cc32
0373/95	1995	B	118	cc32
0374/95	1995	C	11	cc11
0378/95	1995	B	11	cc11
0387/95	1995	C	11	cc11
0392/95	1995	B	32	cc32
0401/95	1995	C	11	cc11
0434/95	1995	C	11	cc11
0436/95	1995	C	11	cc11
0439/95	1995	B	32	cc32
0443/95	1995	C	11	cc11
0444/95	1995	C	11	cc11
0449/95	1995	Y	92	cc92
0450/95	1995	C	11	cc11
0451/95	1995	C	11	cc11
0452/95	1995	C	11	cc11
0463/95	1995	C	2433	cc41/44
0476/95	1995	C	11	cc11
0482/95	1995	B	225	cc865
0483/95	1995	C	11	cc11
0484/95	1995	B	153	cc8
0485/95	1995	C	11	cc11
0495/95	1995	C	11	cc11
0496/95	1995	C	11	cc11
0497/95	1995	C	11	cc11
0499/95	1995	C	11	cc11
0507/95	1995	C	5121	cc11
0508/95	1995	C	11	cc11
0509/95	1995	C	11	cc11
0510/95	1995	C	11	cc11
0511/95	1995	C	11	cc11
0512/95	1995	C	11	cc11
0513/95	1995	C	11	cc11
0514/95	1995	C	11	cc11
0519/95	1995	C	11	cc11
0002/96	1995	C	11	cc11
0005/96	1995	C	11	cc11
0006/96	1996	B	2766	ccUA
0008/96	1996	Y	130	ccUA
0011/96	1996	B	32	cc32
0012/96	1996	C	11	cc11
0014/96	1996	C	11	cc11

0015/96	1996	C	11	cc11
0017/96	1996	C	11	cc11
0019/96	1996	C	11	cc11
0021/96	1996	C	11	cc11
0022/96	1996	B	359	cc41/44
0028/96	1996	B	32	cc32
0029/96	1996	B	101	ccUA
0040/96	1996	C	11	cc11
0086/96	1996	C	11	cc11
0088/96	1996	C	11	cc11
0090/96	1996	C	11	cc11
0093/96	1996	C	11	cc11
0106/96	1996	C	11	cc11
0120/96	1996	C	11	cc11
0154/96	1996	C	11	cc11
0157/96	1996	C	11	cc11
0165/96	1996	C	11	cc11
0170/96	1996	C	11	cc11
0172/96	1996	C	11	cc11
0175/96	1996	C	678	ccUA
0178/96	1996	C	11	cc11
0181/96	1996	C	11	cc11
0187/96	1996	C	11	cc11
0191/96	1996	C	11	cc11
0195/96	1996	B	144	ccUA
0200/96	1996	C	11	cc11
0205/96	1996	C	11	cc11
0208/96	1996	C	11	cc11
0215/96	1996	C	11	cc11
0216/96	1996	C	11	cc11
0220/96	1996	B	225	cc865
0221/96	1996	C	11	cc11
0223/96	1996	B	225	cc865
0224/96	1996	C	11	cc11
0227/96	1996	C	11	cc11
0229/96	1996	C	11	cc11
0238/96	1996	C	11	cc11
0241/96	1996	C	231	cc231
0243/96	1996	C	11	cc11
0254/96	1996	C	213	cc213
0256/96	1996	C	11	cc11
0260/96	1996	C	11	cc11
0263/96	1996	C	66	cc8
0269/96	1996	B	145	cc18
0270/96	1996	C	11	cc11
0284/96	1996	C	11	cc11
0286/96	1996	B	213	cc213
0287/96	1996	C	11	cc11
0290/96	1996	C	11	cc11
0301/96	1996	C	11	cc11
0306/96	1996	C	11	cc11
0312/96	1996	C	11	cc11

0315/96	1996	C	11	cc11
0322/96	1996	C	11	cc11
0335/96	1996	C	11	cc11
0336/96	1996	C	11	cc11
0341/96	1996	C	11	cc11
0344/96	1996	C	11	cc11
0423/96	1996	C	11	cc11
0425/96	1996	C	11	cc11
0435/96	1996	B	18	cc18
0441/96	1996	B	130	ccUA
0457/96	1996	C	11	cc11
0460/96	1996	C	11	cc11
0461/96	1996	C	11	cc11
0464/96	1996	C	11	cc11
0468/96	1996	C	678	ccUA
0490/96	1996	B	44	cc41/44
0493/96	1996	C	11	cc11
0499/96	1997	C	11	cc11
0500/96	1997	C	11	cc11
0503/96	1997	C	11	cc11
0505/96	1997	C	11	cc11
0003/97	1997	B	352	cc269
0006/97	1997	C	11	cc11
0007/97	1997	B	147	ccUA
0013/97	1997	C	11	cc11
0041/97	1997	B	6086	ccUA
0042/97	1997	C	11	cc11
0045/97	1997	C	11	cc11
0046/97	1997	C	11	cc11
0048/97	1997	C	11	cc11
0050/97	1997	C	11	cc11
0051/97	1997	C	11	cc11
0053/97	1997	B	1377	ccUA
0054/97	1997	B	142	cc41/44
0055/97	1997	B	4765	cc18
0061/97	1997	B	1015	cc32
0063/97	1997	C	11	cc11
0069/97	1997	B	269	cc269
0074/97	1997	C	11	cc11
0080/97	1997	B	1274	ccUA
0081/97	1997	C	11	cc11
0082/97	1997	C	11	cc11
0085/97	1997	C	11	cc11
0086/97	1997	C	101	ccUA
0093/97	1997	C	11	cc11
0094/97	1997	C	11	cc11
0097/97	1997	C	11	cc11
0099/97	1997	B	260	cc41/44
0104/97	1997	B	110	cc41/44
0113/97	1997	C	11	cc11
0114/97	1997	C	11	cc11
0121/97	1997	C	11	cc11

0124/97	1997	C	11	cc11
0129/97	1997	C	11	cc11
0130/97	1997	C	11	cc11
0141/97	1997	B	32	cc32
0150/97	1997	B	136	cc41/44
0152/97	1997	B	145	cc18
0154/97	1997	C	11	cc11
0157/97	1997	B	998	cc269
0176/97	1997	C	11	cc11
0177/97	1997	C	11	cc11
0179/97	1997	C	11	cc11
0180/97	1997	C	11	cc11
0181/97	1997	C	11	cc11
0182/97	1997	C	11	cc11
0183/97	1997	C	11	cc11
0185/97	1997	C	11	cc11
0186/97	1997	C	11	cc11
0187/97	1997	C	11	cc11
0196/97	1997	C	678	ccUA
0197/97	1997	C	11	cc11
0198/97	1997	C	11	cc11
0200/97	1997	B	225	cc865
0201/97	1997	C	11	cc11
0204/97	1997	C	5127	cc254
0208/97	1997	C	11	cc11
0209/97	1997	C	11	cc11
0215/97	1997	B	4766	cc292
0221/97	1997	B	269	cc269
0223/97	1997	B	4955	ccUA
0225/97	1997	B	120	ccUA
0227/97	1997	B	1015	cc32
0260/97	1997	C	11	cc11
0265/97	1997	C	11	cc11
0267/97	1997	C	11	cc11
0269/97	1997	C	11	cc11
0277/97	1997	B	118	cc32
0279/97	1997	C	11	cc11
0280/97	1997	C	11	cc11
0281/97	1997	B	18	cc18
0282/97	1997	B	1836	ccUA
0288/97	1997	B	11	cc11
0290/97	1997	C	11	cc11
0295/97	1997	C	11	cc11
0296/97	1997	C	11	cc11
0297/97	1997	C	11	cc11
0298/97	1997	C	11	cc11
0304/97	1997	B	32	cc32
0308/97	1997	C	6087	ccUA
0311/97	1997	C	11	cc11
0313/97	1997	B	32	cc32
0315/97	1997	B	225	cc865
0318/97	1997	C	11	cc11

0319/97	1997	C	2711	cc8
0322/97	1997	B	4955	ccUA
0329/97	1997	C	11	cc11
0331/97	1997	C	11	cc11
0349/97	1997	C	11	cc11
0001/98	1998	B	669	ccUA
0002/98	1998	C	11	cc11
0003/98	1998	C	11	cc11
0004/98	1998	C	11	cc11
0041/98	1998	C	11	cc11
0045/98	1998	C	11	cc11
0046/98	1998	C	11	cc11
0047/98	1998	C	11	cc11
0052/98	1998	C	11	cc11
0054/98	1998	C	11	cc11
0055/98	1998	C	118	cc32
0057/98	1998	B	32	cc32
0058/98	1998	B	33	cc32
0062/98	1998	C	11	cc11
0063/98	1998	B	103	cc103
0065/98	1998	C	11	cc11
0066/98	1998	B	2177	ccUA
0073/98	1998	C	11	cc11
0075/98	1998	B	145	cc18
0079/98	1998	B	11	cc11
0084/98	1998	B	226	cc226
0093/98	1998	B	116	cc116
0097/98	1998	C	11	cc11
0110/98	1998	C	11	cc11
0115/98	1998	C	11	cc11
0117/98	1998	C	11	cc11
0118/98	1998	B	41	cc41/44
0128/98	1998	C	11	cc11
0130/98	1998	C	11	cc11
0131/98	1998	C	11	cc11
0135/98	1998	B	145	cc18
0137/98	1998	C	11	cc11
0139/98	1998	C	11	cc11
0142/98	1998	C	11	cc11
0143/98	1998	B	32	cc32
0146/98	1998	B	669	ccUA
0147/98	1998	C	11	cc11
0148/98	1998	C	11	cc11
0149/98	1998	B	32	cc32
0150/98	1998	B	33	cc32
0151/98	1998	B	33	cc32
0152/98	1998	C	11	cc11
0153/98	1998	C	11	cc11
0154/98	1998	B	145	cc18
0156/98	1998	C	11	cc11
0158/98	1998	C	11	cc11
0162/98	1998	W	2977	cc174

0164/98	1998	C	11	cc11
0170/98	1998	C	11	cc11
0172/98	1998	B	577	cc41/44
0174/98	1998	C	11	cc11
0203/98	1998	C	11	cc11
0204/98	1998	C	11	cc11
0210/98	1998	C	11	cc11
0213/98	1998	C	11	cc11
0214/98	1998	B	18	cc18
0218/98	1998	C	11	cc11
0223/98	1998	C	11	cc11
0224/98	1998	B	1015	cc32
0225/98	1998	C	11	cc11
0284/98	1999	C	8	cc8
0001/99	1999	B	269	cc269
0002/99	1999	B	269	cc269
0003/99	1999	B	35	cc35
0008/99	1999	C	11	cc11
0010/99	1999	C	11	cc11
0012/99	1999	B	118	cc32
0015/99	1999	C	11	cc11
0016/99	1999	C	11	cc11
0019/99	1999	B	33	cc32
0020/99	1999	C	8	cc8
0022/99	1999	B	18	cc18
0024/99	1999	B	269	cc269
0027/99	1999	C	11	cc11
0028/99	1999	C	11	cc11
0030/99	1999	B	1015	cc32
0033/99	1999	B	2177	ccUA
0034/99	1999	B	117	ccUA
0037/99	1999	B	1001	cc18
0038/99	1999	C	23	cc23
0107/99	1999	B	939	ccUA
0108/99	1999	C	8	cc8
0109/99	1999	C	8	cc8
0110/99	1999	B	145	cc18
0111/99	1999	C	11	cc11
0112/99	1999	C	11	cc11
0113/99	1999	C	11	cc11
0115/99	1999	C	11	cc11
0116/99	1999	B	118	cc32
0126/99	1999	C	11	cc11
0131/99	1999	C	8	cc8
0144/99	1999	C	11	cc11
0147/99	1999	B	239	ccUA
0162/99	1999	C	11	cc11
0166/99	1999	C	11	cc11
0171/99	1999	B	269	cc269
0173/99	1999	B	41	cc41/44
0178/99	1999	C	11	cc11
0179/99	1999	C	11	cc11

0182/99	1999	C	11	cc11
0189/99	1999	C	11	cc11
0190/99	1999	C	11	cc11
0191/99	1999	C	11	cc11
0195/99	1999	C	11	cc11
0196/99	1999	C	11	cc11
0197/99	1999	C	11	cc11
0201/99	1999	B	269	cc269
0202/99	1999	B	1015	cc32
0206/99	1999	B	659	cc18
0208/99	1999	B	875	ccUA
0214/99	1999	B	269	cc269
0215/99	1999	C	11	cc11
0220/99	1999	C	11	cc11
0223/99	1999	B	2990	cc41/44
0228/99	1999	C	11	cc11
0231/99	1999	C	11	cc11
0235/99	1999	C	11	cc11
0240/99	1999	C	11	cc11
0241/99	1999	C	11	cc11
0242/99	1999	B	171	cc92
0243/99	1999	C	1260	cc103
0245/99	1999	B	269	cc269
0247/99	1999	C	11	cc11
0255/99	1999	B	145	cc18
0256/99	1999	B	4798	cc18
0263/99	1999	B	33	cc32
0264/99	1999	Y	1625	cc23
0265/99	1999	B	33	cc32
0275/99	1999	B	33	cc32
0276/99	1999	C	11	cc11
0277/99	1999	B	1015	cc32
0278/99	1999	C	11	cc11
0280/99	1999	C	11	cc11
0285/99	1999	B	40	cc41/44
0286/99	1999	B	136	cc41/44
0288/99	1999	B	112	cc41/44
0147/01	2001	B	18	cc18
0218/01	2001	B	145	cc18
0219/01	2001	C	11	cc11
0221/01	2001	C	11	cc11
0010/03	2003	C	11	cc11
0012/03	2003	C	11	cc11
0019/03	2003	C	11	cc11
0039/03	2003	W	11	cc11
0040/03	2003	C	11	cc11
0041/03	2003	C	11	cc11
0065/03	2003	C	11	cc11
0078/03	2003	C	11	cc11
0083/03	2003	C	11	cc11
0091/03	2003	C	11	cc11
0107/03	2003	C	11	cc11

0147/03	2003	C	11	cc11
0149/03	2003	C	11	cc11
0150/03	2003	C	11	cc11
0151/03	2003	C	11	cc11
0175/03	2003	C	11	cc11
0180/03	2003	C	11	cc11
0183/03	2003	C	11	cc11
0188/03	2003	C	11	cc11
0018/04	2004	C	11	cc11
0062/04	2004	C	11	cc11
0064/04	2004	C	11	cc11
0074/04	2004	C	11	cc11
0088/04	2004	C	11	cc11
0108/04	2004	C	11	cc11
0110/04	2004	C	11	cc11
0138/04	2004	C	11	cc11
0183/04	2004	C	11	cc11
0193/04	2004	C	11	cc11
0199/04	2004	C	11	cc11
0278/04	2004	C	11	cc11
0077/07	2007	C	11	cc11
0025/10	2010	C	11	cc11
0010/11	2011	B	112	cc41/44
0083/11	2011	B	5002	cc41/44
0040/13	2013	B	467	cc269
